# Microglial Progranulin: Involvement in Alzheimer’s Disease and Neurodegenerative Diseases

**DOI:** 10.3390/cells8030230

**Published:** 2019-03-11

**Authors:** Anarmaa Mendsaikhan, Ikuo Tooyama, Douglas G. Walker

**Affiliations:** Molecular Neuroscience Research Center, Shiga University of Medical Science, Otsu 520-2192, Japan; kinchan@belle.shiga-med.ac.jp (I.T.); walkerdg@gmail.com (D.G.W.)

**Keywords:** neuroinflammation, growth factor, anti-inflammatory, mutation, amyloid, neurodegeneration

## Abstract

Neurodegenerative diseases such as Alzheimer’s disease have proven resistant to new treatments. The complexity of neurodegenerative disease mechanisms can be highlighted by accumulating evidence for a role for a growth factor, progranulin (PGRN). PGRN is a glycoprotein encoded by the GRN/Grn gene with multiple cellular functions, including neurotrophic, anti-inflammatory and lysosome regulatory properties. Mutations in the GRN gene can lead to frontotemporal lobar degeneration (FTLD), a cause of dementia, and neuronal ceroid lipofuscinosis (NCL), a lysosomal storage disease. Both diseases are associated with loss of PGRN function resulting, amongst other features, in enhanced microglial neuroinflammation and lysosomal dysfunction. PGRN has also been implicated in Alzheimer’s disease (AD). Unlike FTLD, increased expression of PGRN occurs in brains of human AD cases and AD model mice, particularly in activated microglia. How microglial PGRN might be involved in AD and other neurodegenerative diseases will be discussed. A unifying feature of PGRN in diseases might be its modulation of lysosomal function in neurons and microglia. Many experimental models have focused on consequences of PGRN gene deletion: however, possible outcomes of increasing PGRN on microglial inflammation and neurodegeneration will be discussed. We will also suggest directions for future studies on PGRN and microglia in relation to neurodegenerative diseases.

## 1. Introduction

Alzheimer’s disease (AD) is the major cause of cognitive decline and dementia in the elderly. New treatments aimed at removing or preventing Aβ accumulations have generally been clinically ineffective in terms of significantly preventing loss of cognition, though recent amyloid antibody therapies are showing some encouraging results in early phase trials [1,2,3]. However, there is urgent need for new therapeutic targets for AD and other neurodegenerative diseases; firstly, though greater understanding of the complex disease mechanisms involved is needed [4,5]. Neuroinflammation has long been considered a pathological driver of AD pathology, though anti-inflammatory therapies also have not been effective in halting cognitive decline [6]. In this context is the growth factor progranulin (PGRN), which has significant neurotrophic and anti-inflammatory properties, and appears to be expressed in increased amounts by microglia present in conditions of pathology. The seemingly contradictory situation of increased amounts of anti-inflammatory PGRN in activated microglia associated with pathology will be a central theme of this review. We will consider how microglial PGRN might be involved in different complex pathological processes with the goal of addressing whether PGRN might be a therapeutic target for AD and other neurodegenerative diseases with inflammatory components. There have been some recent reviews on PGRN function in brain in relation to disease and lysosomal function [7,8,9]. Our discussion will focus on microglial PGRN and whether there are functional differences between microglial PGRN and neuronal PGRN. However, to provide appropriate background, both sources of PGRN will need to be discussed. We will not directly consider the studies relating PGRN to neurotrophic function in this article.

To directly quote from a recent significant paper on microglial PGRN, it was stated as a concept that microglial PGRN could be considered as a “brake to suppress excessive microglial activation in the aging brain by facilitating phagocytosis and lysosomal trafficking in microglia” [10]. This theme will be examined here.

## 2. Significance

**Figure 1:**
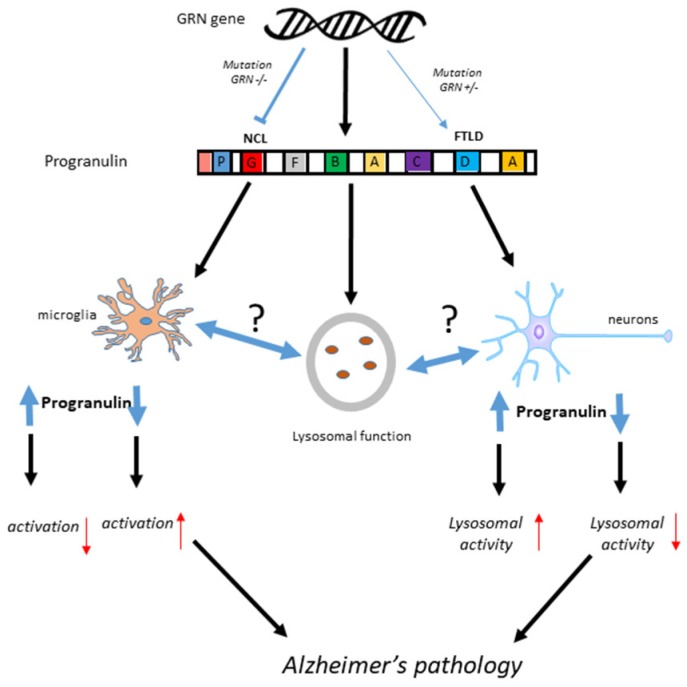
Summary of possible PGRN mechanisms in Alzheimer’s disease, FTLD and NCL. It is hypothesized that loss of PGRN, not increased expression that contribute to Alzheimer’s pathology.

PGRN (also known as epithelin precursor, acrogranin, PC-derived growth factor, GEP, GP88, PEPI, and CLN11) and its granulin cleavage products were first identified in 1992 as growth factors involved in wound healing, vessel growth and cancer [11,12]. The discovery that gene mutations in GRN are linked to frontotemporal dementia (FTD), also described pathologically as frontotemporal lobar degeneration (FTLD) [13,14,15], and to one of the types of the lysosomal storage disease neuronal ceroid lipofuscinosis (NCL) [16,17] inspired many studies on the basic biology of PGRN and its clinical significance. Recently, PGRN deficiency has been associated with Gaucher disease, a lysosomal storage disease that affects many organs and results in significant neurological complications [18]. FTLD can be caused by mutations in a single allele of GRN, while NCL is caused by mutations in both alleles. FTLD is a common cause of early onset dementia in people under 65 years of age [19]. A series of cases from different families with GRN mutations were shown to present various clinical phenotypes; however, all that were examined pathologically had frontotemporal degeneration with accumulations of ubiquitinated TAR DNA-binding protein 43 (TDP-43) positive nuclear and cytoplasmic inclusions [20]. GRN gene mutations resulting in disease invariably are due to the loss of PGRN function. The single nucleotide polymorphism (SNP) rs5848 T allele in the GRN gene has been associated with significant altered risk of developing AD [21,22]. The SNP is present in the 3′ untranslated region of GRN and affects a micro RNA binding site that controls translation of GRN mRNA [23]. It has been speculated that an early downregulation of PGRN might affect the development of AD pathology even if increased amounts of PGRN occur later in disease [24]. Figure 1 provides a summary of the possible interactions of GRN mutations in FTLD and NCL along with how PGRN protein functions could be involved in AD.

## 3. Features of GRN/Grn Gene and Progranulin Protein

### 3.1. GRN/Grn Gene Structure and Regulation

Humans and rodents have a single GRN gene (the abbreviation Grn refers to the rodent gene). The GRN/Grn gene is highly conserved across lower species including insects and fish [25]. The human progranulin gene (GRN) is on 17q21 chromosome and consists of 12 exons. The regulation of GRN gene expression is complex as the gene promoter contains binding sites for a number of different transcription regulators, many of which are activated in inflammation [26]. On account of these different transcription regulatory sequences, studies have identified different factors that can regulate GRN/Grn expression. GRN mRNA expression and PGRN protein levels in a number of myeloid cell lines were most strongly induced by all-trans retinoic acid (vitamin A) and, to lesser extents, by the protein kinase C (PKC) activator phorbol myristate acetate (PMA) and dimethyl sulfoxide (DMSO) [27]. Other studies showed increased PGRN levels in response to estradiol in breast cancer cells [28] and to estrogen in hypothalamus [29]. Inhibiting PKC and phosphatidylinositol 3-kinase (PI3K) diminished PGRN protein amounts in ovarian cancer cell lines, while inhibition of protein kinase A, p38, extracellular signal-regulated kinase and Akt did not affect protein levels [30]. Another complex mechanism involved hypoxia, which induced PGRN mRNA and protein in human neuroblastoma cell lines [31] as a result of activation of the mitogen-activated protein kinases (MAPK)/extracellular signal-regulated kinase (MEK) signaling cascade [31]. Similar findings were demonstrated in the mouse HT-22 neuronal cell line using sodium bisulfite to induce hypoxia [32].

Recently, micro RNAs (miRNA) were demonstrated to regulate PGRN translation. Increased levels of miRNA 29b and miRNA 15/107 reduced protein levels of PGRN in different cell types [33,34]. Inhibiting miRNA 29b resulted in increased PGRN levels, while decreased levels of miRNA15/107 accompanied by increased PGRN levels have been shown in a number of different types of cancers [34]. MiRNA regulation of PGRN may have significance in vivo as 20 different miRNAs showed greatest evidence of dysregulation in frontal cortex of eight FTLD-TDP patients with PGRN mutations compared to 32 FTLD-TDP patients with no apparent genetic abnormalities. These results were validated in frontal cortex for 9 of the 20 miRNAs, with additional measurements showing miRNAs miR-922, miR-516a-3p, miR-571, miR-548b-5p, and miR-548c-5p were also significantly dysregulated in cerebellar tissue samples of GRN mutation carriers [35].

Interleukin (IL)-6 directly induced PGRN levels in liver carcinoma cells [36]; the GRN gene promoter contains IL-6 response element sequences. The GRN gene promoter also contains two possible coordinated lysosomal expression and regulation (CLEAR) sequences. GRN mRNA expression can be regulated by the lysosomal/autophagy master regulator transcription factor-EB (TFEB) that binds to CLEAR sequences [37]. However, alternative mechanisms of regulation of PGRN were identified since cells and mice treated with trehalose, a mammalian Target of Rapamycin (mTOR)-independent activator of autophagy, had elevated levels of PGRN; an effect not dependent on TFEB activation [38].

### 3.2. Progranulin Protein

The human GRN gene codes for a 593 amino acid glycoprotein (PGRN) of 75–80 kDa [39] consisting of highly-conserved 12-cysteine-rich granulin motifs that are tandemly repeated seven and half times [40]. We will mainly be discussing the full-length protein (PGRN), which has the lysosomal and anti-inflammatory properties; however, under certain conditions PGRN can be cleaved by different proteases to produce granulin fragments [41]. In comparison to PGRN, granulins can have a range of pro-inflammatory functions [42]. Based on our ongoing studies of PGRN in human brain tissue samples, we have observed that most PGRN protein appears to be full-length with little detectable low-molecular weight granulin molecules (unpublished observations).

When we consider that many of the reported studies on PGRN used different antibodies for measurements or cellular localization in tissue, it needs to be mentioned that detection of PGRN or granulin in complex tissue can have technical issues that significantly affect results [43]. Many of the available commercial antibodies to PGRN/GRN appear to detect non-specific bands. In addition, detection of PGRN protein by western blot is significantly affected by reducing agents. Omission of reducing agents can increase sensitivity of detection by certain antibodies [43]. Detailed characterization of PGRN antibodies used in all studies is required as many antibodies listed to recognize granulin peptides are likely to also be detecting full length PGRN [43]. This publication provides a valuable resource about the properties of a number of different commercial PGRN antibodies [43].

### 3.3. Progranulin Post-Translational Modification and Secretion

Many of the biological properties of PGRN are dependent on its accumulation in lysosomes, its secretion and endocytosis by neighboring cells and its interactions with other proteins [44,45]. After post-translational modifications in the Golgi complex, a proportion of PGRN remains intracellular by being trafficked to the lysosome. Alternatively, it is secreted from the cell to the extracellular space through the trans-Golgi complex and secretory vacuoles. In the extracellular space, PGRN may be cleaved into granulins by different proteases such as neutrophil elastase [46], matrix metalloproteinase (MMP)-9, MMP-12 [47] and ADAMTS7 [48]. PGRN cleavage is prevented by secretory leukocyte protease inhibitor (SLPI), which binds directly to PGRN linker sequences [49]. In brain cells, the interaction of PGRN, primarily produced by microglia, with SLPI, primarily produced by astrocytes, appears to regulate PGRN cleavage by microglial-expressed MMP-12 [47]. PGRN can also be secreted by neurons in an activity dependent manner in association with brain-derived neurotrophic factor (BDNF). Following treatment for 10 min with 4-aminopyridine or bicucilline to induce neuronal activity, PGRN accumulated in axons. This was diminished with inhibition of neuronal excitation by blocking voltage-gated calcium channels [50]. The majority of circulating or secreted PGRN extracted from GRN-transfected HEK293 cells or from human plasma was a homodimer with molecular weight of 170–180 kDa. This homodimer was resistant to the activity of reducing agents; therefore did not contain disulfide bonds [51]. An alternative mechanism of PGRN secretion has been identified that might be relevant to its function *in vivo*. N-glycosylated PGRN can be secreted in exosomes by human primary skin fibroblast cells and neuronal cells. Significantly reduced numbers of exosomes were produced from SH-SY5Y cells following GRN silencing or from fibroblast cells derived from FTLD cases with PGRN mutations [52].

## 4. Cell Biology of Progranulin

### 4.1. Types of Cells Expressing Progranulin

A wide variety of cells express GRN mRNA and/or PGRN protein, both cell lines cultured in vitro and different types of cells within tissues (www.proteinatlas.org/ENSG00000030582-GRN/cell). Studies identified PGRN expression in epithelial cells, endothelial cells, immune cells and neurons [27,53,54,55]. In rat tissue, mRNA for Grn was highest in spleen and certain endocrine tissues. In kidney, expression was limited to tubule epithelial cells; in spleen it appeared restricted to lymphocytes. Although expression in many different cell lines has been detected, Grn expression in tissue appeared more restricted [56]. PGRN is highly expressed and secreted by myeloid cells in vitro [27], where it can regulate different features of immune function [57,58,59,60,61]. PGRN was highly expressed in foam cell macrophages in atherosclerotic lesions. In mice fed a high fat diet to induce atherosclerosis, those with Grn deletion combined with apolipoprotein E (apoE) knockout had enhanced atherosclerotic lesions compared to apoE knockout mice with intact Grn genes [61]. Loss of PGRN resulted in increased inflammatory profiles, including increased accumulation of cholesterol by macrophages [61].

### 4.2. Progranulin Expression in Human Brain-Derived Cells

An important in vitro study of PGRN expression in human brain-derived cells showed that microglia express and secrete significantly greater amounts of PGRN than astrocytes [47]. Of note were the different mechanisms of regulation of PGRN in microglia compared to astrocytes. PGRN expression and secretion by microglia was downregulated by proinflammatory stimuli (lipopolysaccharide (LPS) or cytokines IL-1β/interferon-gamma (IFN-γ)) but upregulated by IL-4 and IL-13, anti-inflammatory cytokines associated with alternative activation macrophage/microglia phenotypes. By comparison, PGRN expression by astrocytes was stimulated by pro-inflammatory agents, including cytokines IL-1β/IFN-γ or the toll-like receptor 3 ligand polyinosinic:polycytidylic acid (poly I:C) [47].

### 4.3. Expression of Progranulin by Brain Cells in Vivo

Studies of Grn mRNA expression by in situ hybridization in mice showed expression only in classes of neurons (cerebellar Purkinje neurons, hippocampus pyramidal neurons and certain cortical neurons) [54]. A further study using Grn knock-in mice showed expression by immunocytochemistry in neurons and microglia, being particularly strong in neurons of cortex, hippocampus (CA1 and CA3 but weaker in dentate gyrus), thalamus, and hypothalamus and less intense in brain stem, including substantia nigra and cerebellar Purkinje neurons [62]. Neuronal PGRN expression increased with their maturation and age, while microglial expression was dependent on their activation state [62]. Neurotoxic injection of quinolinic acid into striatum resulted in a large increase in the expression of PGRN in microglia, while this neurotoxic injury resulted in less neuronal PGRN expression [62]. In embryonic brains, PGRN was not expressed by neuronal progenitor cells, only by microglia. There was no evidence of PGRN expression by astrocytes [62]. These findings were confirmed in a further study showing intense neuronal immunoreactivity in cingulate and piriform cortex, hippocampus, amygdala, hypothalamus and cerebellar Purkinje neurons. In this study, an overall decrease in PGRN expression with age was observed [63]. Under normal circumstances, microglial PGRN expression was not prominent; however, in a number of in vivo experimental models, upregulated microglial PGRN expression has been shown after traumatic brain injury (TBI), toxin-induced brain injuries, spinal cord injury and nerve axotomy [37,62,64,65,66] (discussed in Section 5.3). Increased expression of PGRN as a feature of “activated” microglia has been widely reported. However, there does now need to be a more precise definition of “activated” to account for possible PGRN anti-inflammatory properties on microglial phenotypes [67]. Recent studies employing single-cell microglia RNA sequencing showed upregulation of Grn mRNA expression in microglia, derived from AD model mice, that had been characterized as having the “disease-associated microglia” (DAM) phenotype, a recently defined late-activation phenotype associated with microglia that restrict AD type pathology [68,69]. These data on microglial Grn expression can be found in supplemental information for reference [68].

## 5. Involvement of PGRN in Disease Processes

### 5.1. PGRN as A Tumor Promoting Factor

Initial discovery of GRN/PGRN was as a growth factor for different cell types [70]. Increased levels of PGRN have consistently been shown to be associated with increased tumorigenic activity. Recent studies showed how PGRN promoted growth or metastasis in breast, colorectal, lymphoma, bladder, cervical and pancreatic cancers [71,72,73,74,75]. This well-documented feature of cancer promotion will need to be considered in elderly human populations if PGRN supplementation is to be used as treatment for neurodegenerative diseases (see Section 8). 

### 5.2. PGRN as A Biomarker for Neurodegenerative Diseases

Measurements of PGRN levels in biofluids (cerebrospinal fluid (CSF) or serum/plasma) have shown some useful diagnostic and predictive value for AD and FTD though results have not been consistent between studies [76,77]. However, a range of peripheral diseases with inflammatory causes or due to cancer or metabolic conditions also show changes in PGRN biofluid levels. These measures lack disease specificity but can have useful disease predictive value [78,79,80,81,82]. There were increased PGRN CSF levels and microglial expression in tissue from multiple sclerosis cases with relapsing-remitting or progressive disease [83]. As expected, subjects with FTLD-related GRN mutations had significantly lower levels of serum and CSF PGRN [84,85]; these studies showed that such measures can be used to monitor progression of disease not diagnosis.

Earlier studies showed little utility for PGRN levels as a useful diagnostic biomarker for AD [22,77]. However, a more recent study showed PGRN CSF levels correlated significantly with frontal lobe memory functions in AD cases, while Aβ42 CSF levels correlated with temporal lobe memory functions [86]. Another study used a large numbers of CSF samples from early-onset AD subjects with AD-causing mutations from the Dominant Inherited Alzheimer’s Disease Network (DIAN) or subjects with late-onset AD from the Alzheimer’s Disease Neuroimaging Initiative (ADNI) [76]. It showed that in early-onset AD patients with identified mutations, CSF PGRN levels increased over the course of the disease, and even was significantly different from non-carriers 10 years before onset of disease symptoms [76]. Furthermore, in late-onset AD cases, higher CSF PGRN levels were associated with more advanced disease stages and cognitive impairment, and once pathology had commenced correlated with CSF levels of soluble triggering receptor expressed on myeloid cells 2 (TREM2) [76]. Both measurements could reflect increasing degrees of microglial activation with disease progression. Serum PGRN levels were not different between control and AD cases and were reduced in subjects with GRN mutations [87]. There was lack of correlation between serum and CSF PGRN levels in AD, FTLD or ALS cases [88]. Others have suggested that serum/plasma PGRN levels can have disease diagnostic value for undiagnosed and presymptomatic FTLD [89]. CSF PGRN levels, not serum PGRN levels, were reduced in non-GRN mutation FTD cases [90].

### 5.3. Role of PGRN in Inflammation

Increased levels of PGRN have been associated with many acute and chronic inflammatory conditions, including atherosclerosis, bacterial infections, influenza infection of lung, human immunodeficiency virus infections, rheumatoid arthritis (RA), amongst others [57,59,91,92,93,94]. Mechanisms of how PGRN can regulate inflammation are still undefined. Certain studies concluded that the central mechanism of inhibiting inflammation was the binding and antagonism by PGRN of the tumor necrosis factor receptor (TNFR)-1 and TNFR-2 [95,96], thus blocking the action of the proinflammatory cytokine tumor necrosis factor (TNF-α). However, other studies have not replicated these findings [97,98]; this issue is still unresolved [99]. It has been shown that PGRN can block the downstream activity of TNF-α by inhibiting the expression and release of CXCL9, CXCL10 or IL-10 [58,100]. It had been speculated that if PGRN could function by blocking TNF-α activation, it would provide a rationale for the use of PGRN in diseases involving excess TNF-α including RA [95]. Increased levels of PGRN have been detected in serum of RA subjects compared to controls [101,102]. Of particular interest in relation to inflammation are the following molecules that can interact with PGRN; Toll-like receptor (TLR)-9 [103], sortilin [44,104], receptor tyrosine kinase receptor EphA2 [105] and prosaposin [106]. These interactions may modulate the anti-inflammatory properties of PGRN in immune cells.

### 5.4. Increased Expression of Microglial Progranulin in Acute Injury Animal Models

Induction of TBI in mice using a stab wound model resulted in significant increase in PGRN immunoreactivity in microglia, which was primarily localized to lysosomes and demonstrated by its colocalization with lysosomal-associated membrane protein (LAMP)-1 [37]. Also, the intensity of CD68 immunoreactivity in microglia was significantly higher in injured mice with Grn gene deletions. These animals showed significantly higher amounts of activated TFEB in microglial nuclei, enhanced expression of many lysosomal genes and significantly greater neuronal loss. This study showed how microglial PGRN regulated inflammatory activation through reducing excessive lysosomal activation [37]. Using the dopaminergic toxin MPTP to create a model of Parkinson’s disease (PD), increased neuronal cell loss and numbers of activated microglia were seen in the substantia nigra pars compacta (SNpc) of toxin-treated mice with Grn gene deletion compared to toxin-treated control mice [65]. It was demonstrated that the enhanced neuronal loss was due to the increased microgliosis, not due to Grn-deficient SNpc neurons being more susceptible to MPTP. In culture, microglia derived from Grn gene deletion mice became hyperactivated in response to proinflammatory stimuli (LPS/IFN-γ); these enhanced responses were diminished in mice with one Grn gene [65]. In mice subjected to spinal cord contusion injury, a large increase in expression of PGRN was detected in activated microglia/macrophages around the sites of injury, while uninjured mice showed very low levels of spinal cord PGRN expression [64]. Using a sciatic axotomy model of nerve injury, neuronal PGRN was decreased and microglial PGRN increased at sites of nerve damage [107]. In this model, an increase in TDP-43 deposits occurred in damaged neurons that coincided with loss of neuronal PGRN. A rapid protective effect of PGRN was seen in a cerebral ischemia reperfusion model using middle cerebral artery occlusion (MCAO) [108]. A dose-dependent effect of PGRN in reducing infarct volume and brain swelling was observed when recombinant PGRN was administered intracerebroventricularly 2 h after MCAO surgery. In this model, there was a rapid decrease of PGRN in mice immediately following MCAO. The consequences of PGRN administration were increased survival of treated animals and reduced neutrophil infiltration into ischemic areas. This study found PGRN administration reduced NF-κB activation and secretion of TNF-α and MMP-12 [108]. In animals treated with pilocarpine to induce status epilepticus, there was increased expression of PGRN in neurons and microglia [60]. Administration of PGRN to pilocarpine-treated animals resulted in significantly increased numbers of microglia at sites of injury.

### 5.5. Animal Models of FTLD Due to GRN Deletion

The first mouse model of FTLD with deletion of both Grn genes showed elevated levels of anxiety and reduced mRNA for the serotonergic receptor 5HT-1A [109]. Other Grn deletion mice models of FTLD have shown age-associated progressive increase of ubiquitin-positive cytoplasmic aggregates of lipofuscin in hippocampal, midbrain, brainstem and thalamic neurons [110]. These lipofuscin accumulations were seen in aged wild type mice with no Grn deletions and in mice with a single Grn gene, but in accelerated amounts in Grn gene deletion mice particularly at late age. Lipofuscin accumulations became significantly increased by 7 months in mice with Grn gene deletion compared to wild type and Grn (+/−) mice. At 12 and 23 months, these gene deletion mice showed significantly increased numbers of activated microglia, reactive astrocytes, neuronal vacuolation, and neuronal loss, as well as ubiquitin positive cytoplasmic inclusions [110]. It was noticeable that there were no differences in these markers between wild type mice and mice with a single Grn gene. It was suggested that lipofuscin accumulations occur due to PGRN deficiency-induced dysfunction of endosomal and lysosomal function, which will further inhibit cellular degradation of protein aggregates in lysosomes [110].

Grn deletion resulted in increased expression of many microglial genes, a feature that increased significantly with the age of the mice [10]. Similar to other studies, PGRN loss resulted in lysosomal defects in neurons and microglia, with one pathological consequence being increased synaptic pruning by microglia in the inhibitory synapses of the ventral thalamus. Microglia from Grn deletion mice showed significantly higher numbers and areas of lysosomes combined with increased CD68, sortilin and LAMP1 immunoreactivity [10]. Grn deletion resulted in increased expression of complement system genes, particularly C1qa. The magnitude of increased C1qa mRNA expression in Grn deletion mice was brain-region specific being lowest in the cortex and highest in the thalamus. There was increased expression of C1qa and C3 mRNA in the thalamus of Grn deletion mice from 4–8 months and this increased dramatically from 12–18 months of age. Increased amounts of C1q and activation fragments of C3 were detected in synaptosomes prepared from Grn deletion mice compared to synaptosomes from Grn wild type mice. Significantly reduced densities of synaptophysin-positive inhibitory synapses were seen in the ventral thalamus of Grn deletion mice at 4 months, a difference that increased with age. It was demonstrated that the increased synaptic pruning was mediated by C1qa depositions and possibly complement activation. In vitro neuron-microglia coculture experiments employing microglia from Grn/C1qa deletion mice and in vivo studies with Grn/C1qa deletion mice did not show the increased synaptic pruning observed in Grn deletion mice. The magnitude of increase in C1qa and C3 mRNA expression in the ventral thalamus of Grn deletion mice correlated with the amount of synaptic pruning. Loss of inhibitory synapses in the ventral thalamus resulted in various behavioral changes, including excessive grooming, while there was significant functional recovery in C1qa/Grn deletion mice compared to Grn deletion mice. Increased numbers of activated microglia and C1qa immunoreactivity were also observed in human FTLD GRN mutation carriers but not in AD cases [10]. Increased microgliosis has been a consistently observed feature in different Grn deletion mice models [111,112], which established that PGRN deficiency can lead directly or indirectly to increased microglial activation.

### 5.6. Progranulin in Other Animal Disease Models

In a mouse model of motor neuron disease/amyotrophic lateral sclerosis (ALS) due to overexpression of mutant superoxide dismutase (SOD)-1, significantly increased levels of Grn mRNA and PGRN protein were detected in spinal cord samples after disease symptoms had started [113]. Similar findings were observed in mice with overexpression of vascular endothelial growth factor (VEGF) and mutated tau, which recapitulate the motor neuron-like disease observed in SOD-1 mutant mice. Based on immunocytochemistry, most of the increased PGRN was due to increased expression by microglia at sites of injury. It was shown that increased amounts of PGRN could reduce the levels of degenerative pathology and neuronal deposits of TDP-43 in TDP-43 mutant mice being used as a model for ALS [66]. Increased numbers of TDP-43 deposits are the hallmark neuropathological feature of human FTLD associated with GRN mutations [107].

### 5.7. Manipulation of Microglial Progranulin Compared to Neuronal Progranulin

To investigate differences between microglial PGRN and neuronal PGRN, several recent studies employed Cre-based conditional gene knockout mice models that allow the selective inhibition of PGRN in particular cell types. Induced deficiency of microglial PGRN employed mice expressing Cre recombinase knocked in to the Lys2 locus (LysMCre) crossed with Grn ^flox/flox^ mice [114], while induced deficiency of neuronal PGRN employed Cre recombinase knocked in on the CaMKII locus (CaMKII-Cre) crossed with Grn ^flox/flox^ mice [115]. Selective depletion of microglial PGRN in mice with normal levels of neuronal PGRN did not cause lipofuscinosis or enhanced neuroinflammation. In addition, these mice with microglial Grn knockout did not have significantly enhanced astrogliosis or microgliosis (except in the hippocampus) compared to controls, features observed in mice with Grn gene deletion [114]. Furthermore, in vitro induced PGRN deficient microglia isolated from brains with normal levels of neuronal PGRN did not show the activated phenotype seen in microglia isolated from Grn gene deletion mice [114]. This finding suggests functional crosstalk might be occurring between PGRN-expressing cells with neuronal PGRN being able to replace microglial PGRN. This would suggest that secreted neuronal PGRN can be endocytosed and trafficked by microglia to restore lost functions.

Restoring neuronal PGRN in Grn haploinsufficient (Grn +/−) mice using an adenovirus vector with Grn linked to a neuronal specific promoter was sufficient to restore lysosomal activity [116]. In a related study, reducing microglial PGRN in mice with Cre-induced neuronal PGRN deficiency did not significantly alter the total brain levels of PGRN, while depletion of neuronal PGRN resulted in 50% decrease in total PGRN levels in frontal cortex and 20–25% in hippocampus [115]. In this mouse model, reduction of microglial PGRN, neuronal PGRN or both did not cause lipofuscinosis in frontal cortex, hippocampus or ventral thalamus, the widely observed feature in GRN deletion mice. Total brain PGRN protein levels in microglial/neuronal PGRN knockdown mice were only reduced to 50% of levels in wild type mice, indicating that either the knockdown was not efficient or that other cellular sources of PGRN were present [115]. Interestingly, although there were no noticeable differences in pathology between mice with microglial compared to neuronal PGRN deficiency, there were some differences in behavioral changes. Mice with microglial PGRN deficiency exhibited enhanced grooming and altered sociability behavior compared to neuronal PGRN deficiency. Surprisingly, deficiency of both microglial and neuronal PGRN restored behavioral differences to control levels [115].

The findings that knockdown of microglial PGRN have limited pathological consequences seem surprising and might indicate incomplete gene deletion. The Cre-recombinase constructs to target cell-specific gene deficiency have limitations. In particular, LysM-Cre constructs to induce knockout of microglia have identified technical concerns about the reliability, efficiency and selectivity of this method, therefore the validity of conclusions from these studies have to be critically examined [117,118]. This microglial-specific Cre construct also results in loss of the lysozyme-2 protein, which can have effects on microglial phenotypes. An alternative microglial-specific construct employs the fractalkine receptor (Cx3Cr1-Cre) [119]. There are other approaches available that selectively remove all microglia from animal brains. The use of antagonists for colony stimulating factor-1 receptor (CSF-1R) to selectively remove all microglia from brain would provide an alternative method to address this issue of microglial PGRN depletion. This technique has been used in multiple studies and could be used to address the issue of how much PGRN in brain is contributed by microglia compared to neuronal PGRN [120,121]. However, it also has some potential limitations as CSF-1R expression has been identified on certain injured neurons [122]

## 6. Role of PGRN in Alzheimer’s Disease

**Figure 2:**
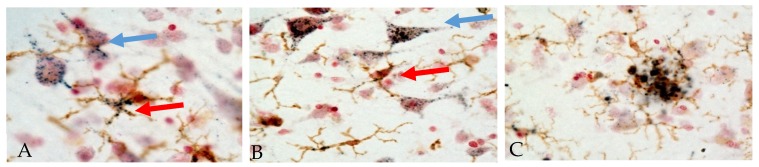
Immunohistochemical localization of PGRN in human temporal cortex. Sections were double-stained with antibody to PGRN (R&D Systems, AF2420) (purple) and the microglia marker IBA-1 (Wako, 019-19741) (brown). The patterns of staining in neurons (blue arrows) and microglia (red arrows) in cells with positive staining for IBA-1 can be seen (Panels A and B). Panel C shows accumulation of microglia (brown) with extracellular PGRN deposits around an amyloid plaque. (**A**) Low pathology control case. (**B**) High pathology control case. (**C**) Alzheimer’s disease case. Staining patterns suggest localization to intracellular vacuoles though plaque staining patterns suggest extracellular aggregates of PGRN.

It has been concluded that a significant role for PGRN in neurodegenerative disease pathology is due to changes in the regulation of microglial inflammatory responses. Examining PGRN cellular localization in human brains showed the following features. Figure 2 shows representative micrographs demonstrating immunolocalization of PGRN in aged human brain. Both neuronal and microglial immunostaining can be seen in this figure (Figure 2A,B). 

Another identified feature is PGRN deposits in association with Aβ plaques (Figure 2, panel C, Mendsaikhan et al., manuscript in preparation [123]). These features have been commented on in previous papers [14,123,124,125,126] though the origin of plaque-associated PGRN is unclear. Initial papers observed features of PGRN immunoreactivity in microglia around senile plaques in AD cases when comparison was being made with neuropathological features of FTLD due to GRN mutations [13,127]. One recent study demonstrated significantly increased levels of PGRN in brain tissue from advanced AD compared to controls using ELISA methodology to quantify protein levels [24]. Increased PGRN mRNA and protein levels have also been measured in AD subjects in a separate study [123]. Accumulations of PGRN immunoreactive structures around Aβ plaques have also been observed in AD model mice [124,125].

A recent study showed that the GRN SNP rs5848 T allele, which results in reduced PGRN expression and increased AD risk, did not have an effect on positron emission tomography detected amyloid image load or cerebrospinal fluid (CSF) Aβ levels but was associated with increased CSF tau levels [128]. A large series of subjects with the SNP rs5848 T allele had reduced plasma levels of PGRN and reduced GRN mRNA expression in AD subjects but not controls, however these results had marginal statistical significance [22].

### 6.1. PGRN in Animal Models of Alzheimer’s Disease

Most studies have relied on mice models to investigate possible involvement of PGRN in AD pathogenesis. As a consequence of using different AD mouse and Grn deletion models, there have been some conflicting data on the consequences of PGRN deficiency on AD-type pathology. In 16-month-old APP/PS1/Grn-/- mice, there was significantly reduced plaque deposition, particularly diffuse Aβ plaques, in hippocampus and cortex, but no significant effect on dense-core Thio-S positive plaques [128]. This study also showed that Grn-deficient mice had significantly increased CD68 immunoreactivity, particularly in microglia associated with plaques. APP/PS1 mice with a single Grn gene were not significantly different from APP/PS1 mice with both copies of Grn. Gene expression profiling of 6-month-old Grn-deficient APP/PS1 mice showed higher levels of TYRO protein tyrosine kinase binding protein (TYROBP) network of inflammation-related genes, including TREM-2, TYROBP (DAP12), C1qA, CD68 and CD22, but not higher levels of proinflammatory cytokines IL1β, TNFα or IL6, or inducible nitric oxide synthase compared to APP/PS1 mice [128]. In this study, APP/PS1 Grn-deficient mice had reduced numbers of dystrophic neurites at 16 months and improved spatial memory in the Morris water maze test. PGRN deficiency reduced the spatial learning acquisition deficit at certain time points but not in the probe trial test. As demonstrated in another study [10], this study confirmed that Grn deficiency can lead to an accumulation of microglial-derived complement C1qa on neuronal synapses [128]. A recent small-scale study showed that APP/PS1/Grn +/− mice had marginally significant reduction in plaque numbers and plaque area at 16–18 months [129].

Increased expression of PGRN (8.5-fold) was shown in laser microdissected tissue from Tg2576 mice at 18 months, a model that develops plaques at a slow rate, and also by 1.8-fold in APP/PS1 mice at 10 months, a more rapid plaque developing AD model. Immunohistochemistry for PGRN identified accumulations around Aβ plaques and also expression by neurons and microglia, but not astrocytes [124]. This study included staining of some early onset human AD cases with identified genetic mutations and sporadic late onset AD cases, which showed similar localization of PGRN around dense core plaques as the AD model mice [124]. This study also demonstrated a significant correlation between intensity of PGRN immunoreactivity in cell processes and cell bodies with Aβ load in these AD mouse models.

Measurement of PGRN levels in different AD mouse models showed reduced levels in low plaque developing AD model mice at 11–12 months. Using another strain of mice with high levels of expression of mutated APP (J20 strain), PGRN levels were less than controls at 3 and 7 months before significant plaque development, but at similar levels at 27 months when plaque development was significant. In the aggressive 5× FAD mouse model, which develops plaques at 2 months, there were significantly increased amounts of PGRN by 13 months [24]. In low plaque APP mice with Grn deficiency, there were increased numbers of CD68 positive microglia compared to Grn-deficient mice without APP expression as well as significantly reduced performance in the Morris water maze test. In high APP expressing plaque developing LysM-Cre mice crossed with flox Grn to induce microglial PGRN deficiency, there was increased microgliosis, increased expression of TNFα and IL1α, reduced phagocytosis and increased amounts of plaque deposits compared to APP expressers with Grn genes. In vitro microglia from LysM-Cre/Grn ^flox/flox^ mice showed 25% reduction in bead phagocytosis. This study demonstrated reduction of microglial Grn mRNA by around 50%, highlighting the technical limitations of this technique. Studies on the outcomes of complete loss of microglial PGRN on AD type pathology are needed [24], though the limited loss of microglial PGRN might more accurately represent the features of early AD [23]. By contrast, overexpression of PGRN in microglia, using a lentivirus construct expressing the Grn gene with the macrophage/microglia specific MCSF promoter administered to 5xFAD mice resulted in significant neuroprotection from toxic Aβ, significant protection from hippocampus dependent memory deficits and also significantly reduced hippocampal plaque deposition [24]. In these animals, the amounts of plaque deposition (immunoreactive and Thioflavin-S stained) negatively correlated with PGRN levels. Mice treated with PGRN gene therapy showed significant improvement in behavioral testing [24]. A conclusion from this study using the different types of plaque developing mice was that early loss of PGRN might be one event precipitating the development of AD pathology. This is a hypothesis that needs to be examined with human brain tissues with differing amounts of AD pathology. Overexpression of PGRN by lentivirus gene transduction vectors showed significant chemoattractant activity with increased numbers of microglia at the sites of injection. The chemoattractant properties of PGRN were confirmed in vitro using isolated microglia. This study also demonstrated the effect of increased PGRN on enhancing microglial endocytosis of Aβ fibrils [130].

PGRN deficiency (Grn +/−) in mutant tau P301L tangle forming mice resulted in increased levels of tangle-associated phosphorylated tau (tau phosphorylated at threonine 181 and serine 422). This was demonstrated by western blot and immunohistochemistry and was possibly due to increased activity of cyclin dependent kinases (CDK) [131]. In addition, 16-month-old APP/PS1 Grn-deficient mice showed higher levels of phosphorylated tau at serine 404 compared to APP/PS1 Grn intact mice. Overexpression of mutant P301L tau in Grn-deficient mice by injection of a mutant tau AAV expression vector into the entorhinal cortex resulted in enhanced immunoreactivity demonstrated with antibodies to phosphorylated tau (serine 202 and threonine 205) (antibody AT8) and phosphorylated tau (serine 231) (antibody AT180) [131].

### 6.2. Possible Mechanisms of Action of PGRN in Immune Cells

One question is whether microglial expressed PGRN, which can be detected intracellularly by immunohistochemistry (see Figure 2), mainly in lysosomes, has anti-inflammatory properties or whether PGRN needs to be secreted and/or to bind membrane receptors such as TLR-9 [103], sortilin [44], receptor tyrosine kinase receptor EphA2 [105] or prosaposin (PSAP) for activity [132]. These interacting proteins will affect the properties and intracellular localization of PGRN. RNA profiling data shows sortilin expression in all brain cell types, particularly oligodendrocytes (www.brainrnaseq.org: search term sort1) but immunohistochemistry staining for sortilin is primarily in neurons (www.proteinatlas.org/ENSG00000134243-SORT1/tissue/cerebral+cortex#imid_3598756). A recent paper demonstrated low amounts of sortilin expression by microglia in vitro, with increased amounts on intracellular membranes of Grn deletion microglia [10]. Immunohistochemistry for sortilin was confirmed in an AD mouse model, though extracellular sortilin bound to plaques was a feature only observed in human AD brains [133]. In contrast, EphA2 can be highly expressed in rodent microglia/macrophages and endothelial cells (www.brainrnaseq.org search term epha2). Human brain immunohistochemistry studies for EphA2 have not been reported. The data on PGRN interactions with TLR-9 have not been replicated but the study suggested that PGRN could be a co-receptor for TLR-9 aiding in the binding of TLR-9 ligand unmethylated CpG oligonucleotides (ODN). The physiological significance of this interaction is unclear, but a related series of studies demonstrated how TLR-9 stimulation due to peripheral administration of TLR-9 ligand CpG ODN into AD model mice resulted in significant clearance of Aβ from brain [134]. It has been hypothesized that PGRN might synergize with TLR-9 to promote microglial phagocytosis [134]. Although TLR-9 immunolocalization has not been demonstrated in human brains as its expression levels appear to be very low [135], it has been hypothesized that microglia would be the responding cell type to TLR-9 ligands [134]. PSAP and PGRN have physical interactions through GRN linker sequences and facilitate trafficking of each other to lysosomes through a sortilin-independent pathway [136,137]. Although it has not been shown that human brain microglia express PSAP in vivo, expression of PSAP mRNA in microglia in rat facial nerve after injury has been demonstrated [106]. No increased levels of PSAP were detected by western blot in brain samples from FTLD human cases or Grn-deficient mice, but there were increased levels of saposin D, which is derived from PSAP, in microglia directly isolated from brains of Grn-deficient mice [138,139].

## 7. Progranulin Effects on Lysosomal Function

Evidence of lysosomal dysfunction due to compromised protein degradation was an early observation in brains from cases with FTLD due to GRN mutations and linked PGRN to lysosomal function [140,141]. Homozygous loss of GRN results in profound lysosomal disruption [17]. Although PGRN has been demonstrated to have pleiotropic properties, its effects on lysosomal function may be a unifying mechanistic feature. Lysosomal function is essential for all types of cellular homeostasis, especially in brain. Lysosomal function is also directly connected to microglial activation states and inflammation [142] with lysosomes being required to remove unwanted proteins, such as aggregated Aβ and tau, phagocytosed by microglia. With aging and AD, there is significant evidence of lysosomal/autophagic defects in brain [143]. Accumulations of lipofuscin, a consequence of lysosomal defects, can be seen in many neurons in tissue sections from most aged human brains with increased amounts of lipofuscin and other markers of lysosomal dysfunction being a feature of brains of FTLD cases with GRN mutations [139,144]. Analyses of lysosomal and disease-associated proteins in human cases of FTLD with GRN mutations showed significantly increased biochemical levels of saposin D (but not PSAP), mature cathepsin D, LAMP1, LAMP2, TMEM106B and phosphorylated TDP-43. These observations were supported by immunohistochemical staining of affected tissue sections [139]. Although PGRN can be processed in lysosomes into granulin molecules by enzymes such as MMP12, cathepsin D and cathepsin L [43,145], it is full-length PGRN that has essential roles for maintaining lysosomal function.

Gene expression profiling of Grn deletion mice at 6 months and 12 months demonstrated significant changes in expression of only 8 genes with the largest changes being for CD68, CD63 and LAMP1, all lysosomal membrane proteins [138]. In addition, there was significantly increased expression of lysosomal hydrolases hexosaminidase b (Hexb) and cathepsin D at 12 months in these mice. This study identified expression of many TFEB-regulated lysosome-associated genes but most were unchanged in Grn deletion mice compared to wild type mice. TFEB expression was downregulated in 6-month-old Grn deletion mice but was at control levels in these mice at 12 months. Increased levels of activated forms of cathepsin D, B, and L were also demonstrated in Grn deletion mice with changes being more pronounced in 20-month-old mice. Using fibroblasts derived from Grn deletion mice, enhanced protein degradation was evident and reduced amounts of ubiquitinated proteins and p62, the ubiquitin-adapter protein, compared to controls. Selective changes in cathepsin levels were shown in brain-purified microglia from Grn gene deletion mice, particularly cathepsins D, B and L. Changes in lysosomal proteins in these purified brain microglia occurred by 4 months with significant increases in saposin D and LAMP1. A similar study using purified neurons from Grn deletion mice showed enhanced lysosomal activity [146]. Using TBI to cause brain injury in wild type and Grn gene deletion mice, there was evidence of increased expression of many different lysosomal genes in Grn deletion mice. This was due to excessive activation of TFEB in Grn deletion microglia from TBI injured mice [37] that resulted in enhanced neuronal damage. A recent study used an adeno-associated virus (AAV) Grn expression vector to express PGRN specifically in neurons of Grn deletion mice [147]. A number of beneficial outcomes were observed in these AAV-administered Grn deletion mice including reversal of lipofuscinosis and reduction in microgliosis. These effects were seen even though overexpression of PGRN was very variable across different brain regions. This treatment resulted in reduced lysosomal dysfunction and reduced levels of the microglial lysosomal protein CD68 in hippocampus CA2 and ventral thalamus brain regions, even though there were increased numbers microglia in medial prefrontal cortex. A significant effect was observed even in brain regions where PGRN overexpression was moderate [147].

This group recently extended these finding by showing that PGRN regulates lysosomal function and biogenesis by controlling the acidification of lysosomes [148]. Levels of mature cathepsin D were greater in a lysosomal environment of pH 4. Using the MG6 mouse microglial cell line, increasing the pH of lysosomes increased expression of Grn, Lamp1 and cathepsin D mRNA, an effect that was enhanced in serum-free media. Overexpression of PGRN in a neuronal cell line showed its localization in the endoplasmic reticulum (ER), lysosome and Golgi apparatus. The consequence of this over-expression was reduced cathepsin D, B, K and L/S but not gamma secretase activity. This study also identified through cellular fractionation studies that PGRN was predominantly membrane-associated [148]. The primary lysosomal enzyme changed as a result of PGRN overexpression was cathepsin D, whose level was decreased. Consistent with other studies, microglia from Grn deletion mice had increased LAMP1, Hexa and mature cathepsin D [148].

Another lysosomal protein that is affected by PGRN deficiency is transmembrane protein 106B (TMEM106B), which has been defined as a specific genetic modifier for FTLD due to GRN mutations [149]. TMEM106B has been shown to regulate PGRN levels in FTLD cases [149]. No physical interactions between TMEM106B and PGRN have been reported, but there is strong evidence for their functional interaction [146]. Increased levels of TMEM106B occur with PGRN deficiency in mice, resulting in increased TMEM106B accumulation, enlarged lysosomal morphology, and increased lipofuscinosis. TMEM106B overexpression in Grn-deficient mice resulted in enhanced lipofuscinosis [150]. TMEM106B mRNA levels were significantly decreased in AD cases compared to controls, while Grn mRNA levels were increased [123]. There was a significant negative correlation between these two measures. This study showed strong neuronal staining for TMEM106B with immunoreactivity also being observed in astrocytes, endothelial cells, and microglia. TMEM106B mRNA was also detectable in a human microglial cell line [123]. These promising findings suggest further studies related to TMEM106B, PGRN and neuroinflammation are warranted.

These recent experiments confirm the close link of PGRN to many facets of lysosomal function. There remains the question of how the balance of too much or too little lysosomal activity, which appears to be different between microglia and neurons [148], will be disturbed with PGRN supplementation.

## 8. Is Progranulin a Potential Therapeutic Target for Alzheimer’s Disease, Parkinson’s Disease and FTLD?

This is the central question when considering mechanisms of neurodegeneration; namely does PGRN supplementation have the potential to be a means of disease therapy. Based on results from human FTLD studies or animal models of FTLD involving deletion of Grn genes, it is clear that loss of PGRN function contributes to neurodegeneration. However we are still left with the problems of separating out function of neuronal and microglial PGRN in vivo and determining how they interact in order to decide which cellular source of PGRN should be enhanced.

Supplementation of intracellular PGRN into the brain is possible with gene transduction vectors. and this has shown significant protective effects when used in different models of FTLD. For example, in a recent study, restoring neuronal GRN in haploinsufficient Grn (+/−) mice, which represent a better model for FTLD than Grn deletion mice, using an adenovirus vector expressing Grn was sufficient to restore animals to normal behavior, restore amygdala neuronal function, and stabilize neuronal lysosomal function in prefrontal cortex [116]. In addition to findings discussed in Section 5.1 with AD models, which indicated the therapeutic potential of PGRN therapy for AD [24], the following two papers derive from the biotechnology company “NeuroDyn” that has made PGRN therapeutics a central therapeutic development program. Protective effects using a Grn retrovirus gene delivery mechanism administered into the hippocampus at 8 months were observed in Tg2576 Aβ plaque-developing AD model mice [151]. Animals sacrificed 4 months after gene administration had significantly lower plaque loads in the hippocampus (CA1 and dentate gyrus), entorhinal cortex and frontal cortex, and significantly higher levels of the Aβ-degrading enzyme neprilysin [151]. Treated animals had significantly reduced astrocytosis, microgliosis and synaptic density loss. Overall, these results point to protective properties of PGRN in neurodegenerative diseases even where there is no deficit of PGRN. This group used the same PGRN gene therapy vector to protect dopaminergic neurons in the substantia nigra of a toxin-induced Parkinson’s disease (PD) animal model [152]. Administration of PGRN expressing vector into the substantia nigra of MPTP-treated mice resulted in significant protection from toxicity as shown in locomotor activity tests, protection of SN dopaminergic neurons and maintenance of striatal dopamine levels [152]. The gene delivery method resulted in increased PGRN expression in neurons and glial cells [151,152]. Despite the positive results obtained using Grn transduction vectors for treating experimental models of AD or FTLD, the use of PGRN overexpression needs to be approached with caution. Increased PGRN could promote tumorigenesis, and a recent study has shown that PGRN overexpression mediated by an AAV vector resulted in hippocampal neurodegeneration that was preceded by significant T cell infiltration [153]. In this study, employing a different Grn delivery vector that resulted in increased secretion of PGRN into CSF resulted in ependymal hypertrophy accompanied by T cell infiltration [153].

## 9. Summary

**Figure 3:**
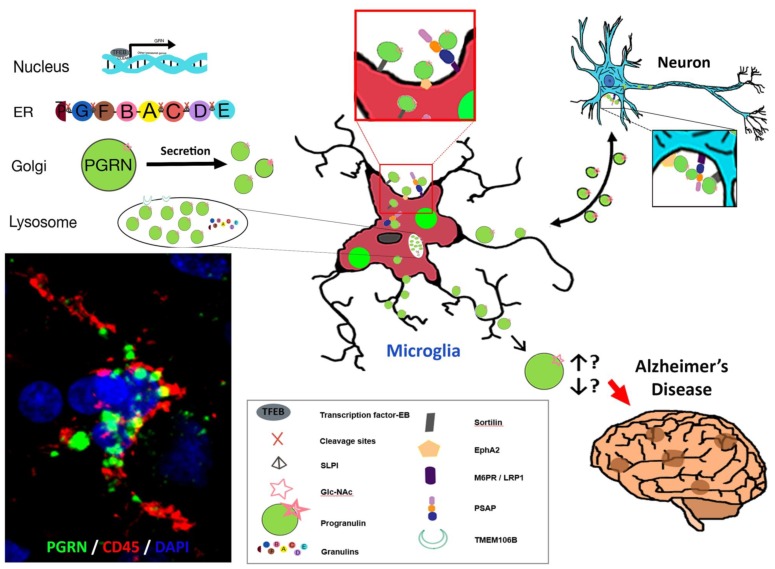
Some Features of PGRN, microglia and Alzheimer’s disease. (**A**) Confocal image of PGRN positive (green) accumulations within a CD45 positive microglia in a human AD brain. (**B**) Intracellular processing of Grn to PGRN and granulins. (**C**) The adjacent diagrams suggests how features identified in vivo and in vitro might interact and be involved in PGRN function in AD. There are unanswered questions about the interaction of proteins with PGRN intracellularly and with secreted PGRN. The direction of interaction of PGRN between microglia and neurons is unresolved. The diagram represents PGRN as accumulations as seen in panel A but is believed to at least be present as dimers. The final unresolved issue is whether excess of PGRN precipitates Alzheimer’s disease pathology or whether it is a deficiency of PGRN or loss of function that has this effect.

Figure 3 summarizes some of the key salient features of PGRN and AD focusing on microglia that we have discussed but also suggests further research questions. In Panel A, the three-color confocal picture represents a single microglia present in an aged brain. This is representative of most brain samples we have examined, both AD and aged, and shows strong accumulations of PGRN (green) within lysosomes and other vacuoles in a CD45-positive microglia (red) (Figure 3A). Published works of PGRN in brain and our figures (Figure 2 and Figure 3) identify PGRN as accumulations within cells. PGRN is known to interact with sortilin, prosaposin, and EphA2 amongst others and found to be in close association with TMEM106B in lysosomes. These include receptors that can mediate endocytosis. These interacting proteins are expressed by neurons and microglia at different levels.

We still do not know what biochemical forms the PGRN in neurons, microglia or plaques takes (dimer, monomer, aggregated, glycosylated, bound to different proteins) and what is the functional state of PGRN positive activated microglia. These features will determine whether “microglial PGRN” that is upregulated at the end stage of disease is slowing or hastening AD pathology by affecting inflammatory and phagocytic functions. Alternatively, we are suggesting that there could possibly be a functional decrease in PGRN in AD due to it being in a non-bioactive (aggregated) state. The ultimate question to address is whether supplementation of PGRN should be focused on neurons or microglia to have a therapeutic effect.

## 10. Conclusions and Future Directions for Studies of PGRN in Alzheimer’s and Parkinson’s Disease and Other Neurodegenerative Diseases

Reviewing the studies relating to PGRN in AD, other neurodegenerative diseases including FTLD, or animal models of these diseases, one must conclude that there are a number of features of PGRN biochemistry and cellular biology that still require investigations before attempting therapeutic treatments in humans. There needs to be caution with interpretation of results from Grn deletion mice studies for relevance to AD. The consequences of complete loss of PGRN are profound but have limited human disease relevance except for subjects with NCL. In AD, we have a situation of excess PGRN without knowing if the molecules have bioactivity. Supplementation of PGRN by gene vectors could be effective as the newly synthesized PGRN might be in a form that has activity for altering microglia or neuron functions. This leads to the question: What is the biochemical nature of pathology-associated PGRN?

### 10.1. Biochemical Nature of Pathology Associated Progranulin

We have discussed the pleiotropic properties of PGRN from various experimental studies but must conclude that as appearance of PGRN associated with plaques appears to be that of aggregated protein, does it lack bioactivity or possibly have antagonistic effects? As mentioned, it has been shown that secreted forms of PGRN appear to exist as a homodimer, but under certain conditions, PGRN can aggregate and form oligomers. This oligomerization of PGRN being regulated by the PGRN-interacting protein PSAP [132]. We have to conclude at this stage that it is unclear whether the PGRN observed in diseased AD brains represent bioactive forms or is it complexed with other proteins. Reduced levels of PSAP result in predominance of monomeric forms of PGRN, while overexpression promotes PGRN oligomerization [132], while reduction of PSAP levels resulted in increased expression and secretion of brain PGRN [132]. PSAP deficiency has been shown in three rare genetic cases to result in a large amount of neuronal loss due to lysosomal deficiencies [154]. There appear to be no reported study describing PSAP distribution in human AD brains. Such a study is needed as it would be informative to determine if PSAP could be colocalized with PGRN in microglia or neurons, or extracellularly on PGRN associated with Aβ plaques in pathologically affected brains. Similarly, studies to determine if there are significant interactions of EphA2 with PGRN in diseased human brains are suggested. Studies are also warranted to determine if PGRN and TMEM106B functional interactions directly affect microglial activation states and resulting properties. These described protein interaction studies can be done with techniques such as immunoprecipitation, proteomics, and multi-color confocal microscopy. These studies should be done with human materials but are dependent on access to consistently high-quality human brain tissue samples.

### 10.2. Phenotyping of Microglia Overexpressing Progranulin

It has been shown in frontal cortex of GRN mutation carriers, contrary to expectations, there was increased expression of GRN mRNA. The extra GRN mRNA was contributed by the increased numbers of activated microglia in this region of pathology even though there was only expression of a single GRN allele [155]. Both in AD and FTLD as well as in other neurodegenerative diseases, it would be worthwhile to characterize the activation phenotypes of microglia that express PGRN in human diseased brains. The only significant in vitro experimental study on expression of PGRN by human microglia showed that it was regulated by anti-inflammatory cytokines IL-4 and IL-13. If this is reflected in vivo, it would suggest that PGRN expressing microglia are not the damaging reactive microglia but have a reparative phenotype. Such studies will require examination of PGRN immunoreactive microglia with other microglial functional markers.

### 10.3. Further Human Brain Studies of PGRN in Other Brain Diseases Involving Microglia

It is now appreciated that many of the features of these uniquely human brain diseases of aging cannot be accurately modeled in rodents. Besides AD and FTLD, if one considers PD, where PGRN supplementation showed significant protective properties in an animal model [152], there have been no published reports of PGRN expression in human PD affected brain tissue. This is surprising as the GRN SNP rs5848 has also been associated with altered risk of developing PD [152,156], and reduced serum PGRN levels have been associated with increased risk of PD [157]. As PD is a neurodegenerative disease also with a significant microglial component in affected brain areas [158,159], such studies of PGRN in human tissues with deposits of aggregated/abnormal α-synuclein could also be productive.

The main aim of this review has been to consider the features of PGRN expressed by microglia in human brains and stimulate further research in this field. PGRN has so many properties, it will be a continuing challenge to identify which are deficient in neurodegenerative diseases such as AD, where there appears to be increased amounts of protein. As mentioned in the introduction, microglial PGRN should suppress proinflammatory microglial activation, but we still have no clear indication that this is happening in AD. Investigating this feature alone makes this aspect of PGRN research potentially significant and rewarding.
